# Synthetic Peptides as Structural Maquettes of Angiotensin-I Converting Enzyme Catalytic Sites

**DOI:** 10.1155/2010/820476

**Published:** 2010-06-09

**Authors:** Zinovia Spyranti, Athanassios S. Galanis, George Pairas, Georgios A. Spyroulias, Evy Manessi-Zoupa, Paul Cordopatis

**Affiliations:** ^1^Department of Pharmacy, University of Patras, GR-26504, Patras, Greece; ^2^Department of Chemistry, University of Patras, GR-26504, Patras, Greece

## Abstract

The rational design of synthetic peptides is proposed as an efficient strategy for the structural investigation of crucial protein domains difficult to be produced. Only after half a century since the function of ACE was first reported, was its crystal structure solved. The main obstacle to be overcome for the determination of the high resolution structure was the crystallization of the highly hydrophobic transmembrane domain. Following our previous work, synthetic peptides and Zinc(II) metal ions are used to build structural maquettes of the two Zn-catalytic active sites of the ACE somatic isoform. Structural investigations of the synthetic peptides, representing the two different somatic isoform active sites, through circular dichroism and NMR experiments are reported.

## 1. Introduction

Angiotensin Converting Enzyme (ACE) catalyses the conversion of angiotensin-I (AI) to the vasoconstrictor angiotensin-II (AII) [1] and inactivates the vasodilatory peptide bradykinin by removing C-terminal dipeptides [2]. The inhibition of ACE enzymatic activity against AI was considered as one of the major challenges against hypertensive disease and congestive heart failure [3]. Over the past 20 years, ACE inhibitors have presented significant cardioprotective and vasculoprotective activity, by reducing oxidative stress and inflammation in the endothelium. Moreover, ACE inhibitors have been effective in improving blood flow and flow-mediated vasodilation. Inhibition of the angiotensin converting enzyme significantly reduces cardiovascular risk in a broad range of high-risk patients [4, 5]. 

ACE is a Zinc Metallopeptidase and one of the major components of the Renin-Angiotensin System (RAS) that regulates blood pressure [6–8]. In human, ACE is expressed as a somatic isoform in endothelial, epithelial, and neuroepithelial cells and as a smaller isoform only in male germinal cells. Somatic ACE (1306 AA, 150 kDa) consists of two homologous domains, one at each terminal, containing zinc catalytic sites (N-and C-zinc catalytic sites) [9]. The testis isoform is composed of 732 residues (83 kDa) with the 665-residue C-terminal domain being identical to the C-terminal domain of the somatic form [10–13]. Both isoforms possess a zinc-binding domain, with the somatic isoform containing one additional high homology active site (N-catalytic site). ACE active sites possess the characteristic HEXXH Zn-binding motif (the two His comprise the first two Zn-ligands) and falls into the gluzincin family [14]. The third Zn ligand, glutamic acid, is sited 23 residues towards the enzyme C-terminal at the second characteristic sequence EAXGD [15]. The fourth zinc ligand is a water molecule.

In subsequent to our previous work [16, 17], novel synthetic peptides have been investigated as structural maquettes, in order to shed further light on the conformational characteristics of both somatic isoform catalytic site domains. 

Design, peptide synthesis, and a thorough investigation of the optimal conditions in order to mimic as closely as possible the structure of the enzyme native active sites are reported. The peptide structures have been determined through circular dichroism and NMR experiments. 

The conformational differences of the peptide maquettes representing the two sACE active sites have been also investigated. Moreover, the NMR solution structures of the peptides are being compared to the crystal structure of somatic ACE_N_ active site [18] and the testis ACE isoform (tACE) [19] that corresponds to the somatic ACE_C_ domain. In general, we report herein a work, which exploits the potential of peptide chemistry to synthesize polypeptides that represent protein domains or functional fragments and applies a step-by-step investigation strategy able to extract crucial structural data, especially for the metal active sites of enzymes/proteins or others biopolymers, whose biological expression and crystallization are difficult to be acquired, such as GPCRs and highly hydrophobic transmembrane proteins.

## 2. Materials and Methods

### 2.1. Peptide Synthesis

**Figure 1:**
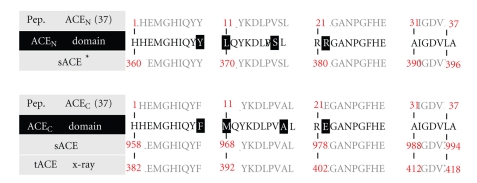
Synthetic peptide maquettes of the N- and C- active site domains of human somatic ACE (sACE). Sequence numbering of peptides, sACE, testis isoform (tACE) and crystal structures domains. The different residues among the two sequences are highlighted.

Both peptides representing the domains of the two somatic ACE (sACE) active sites ACE_N_(37): sACE(360–396); ACE_C_(37): sACE(958–994) were synthesized on solid support by Fmoc/tBu chemistry, as previously described [15]. Sequence enumeration of the synthetic peptides and the corresponding domains of the human somatic isoform as well as the numbering of the C-(tACE) and N-domain (ACE_N_) crystal structures are shown in Figure 1.

### 2.2. Circular Dichroism Experiments

CD experiments were acquired for both ACE_N_(37) and ACE_C_(37) peptides, monitoring the effect of different trifluoroethanol (TFE) concentration, pH values, and Zn^+2^ addition on their conformations. Spectra were recorded on a Jasco 710 spectropolarimeter using quartz cells of 1.0 cm and 0.5 cm path length, in the far-UV (200 nm–260 nm) at a scanning rate of 100 nm/min, a time constant of 1 s, and a bandwidth of 1 nm. Spectral resolution was 0.2 nm, and 4 scans were averaged per spectrum. 

The concentration used for each sample was 0.3–0.35 mg/ml of pure peptide in a buffer of 50 mM Tris-HCl and 200 mM NaCl. The effect of TFE on the peptide conformation was monitored for 0%–100% TFE (v/v), 25°C, and pH = 7.0. Spectra of different pH values ranging from 2.6 to 7.0 were recorded for the Zn-containing ACE_N_(37) peptide, at 65% TFE and 25°C. Quantitative evaluation of secondary structure according to the CD data was calculated using the CDNN CD Spectra Deconvolution Program obtained from http://bioinformatik.biochemtech.unihalle.de/cdnn/ [20]. The CD spectra are reported in molar ellipticity as mdeg × cm^2^/dmol according to molecular masses and peptide length.

### 2.3. Nuclear Magnetic Resonance Experiments

5 mg of peptide samples were dissolved in a mixture of 65% TFE in H_2_O, containing 50 mM Tris buffer and 200 mM NaCl. ZnCl_2_ was added in a slight excess of the peptide equivalents (1:1.1) until 0.5 ml final sample volume, with the peptide concentration being approximately 2 mM at pH value 4.9–5.1. 

Data were acquired at 298 K on a Bruker Avance 600 MHz spectrometer. ^1^H 1D NMR spectra were recorded using spectral width of 12–17 ppm with or without presaturation of the H_2_O signal. ^1^H-^1^H 2D TOCSY [21, 22] were recorded using the MLEV-17 spin lock sequence using *τ*_*m*_ = 80 ms. ^15^N HSQC and ^13^C HSQC spectra [23, 24] have been recorded at 500 MHz equipped with cryoprobe for 15N/13C nuclei in natural abundance. ^1^H-^1^H TPPI NOESY [25, 26] spectra were acquired using mixing time *τ*_*m*_ = 200 ms applying water suppression during the relaxation delay and mixing time. For data processing and spectral analysis, the standard Bruker software (XWIN-NMR 3.5) and XEASY program [27] (ETH, Zurich) were used.

**Figure 2:**
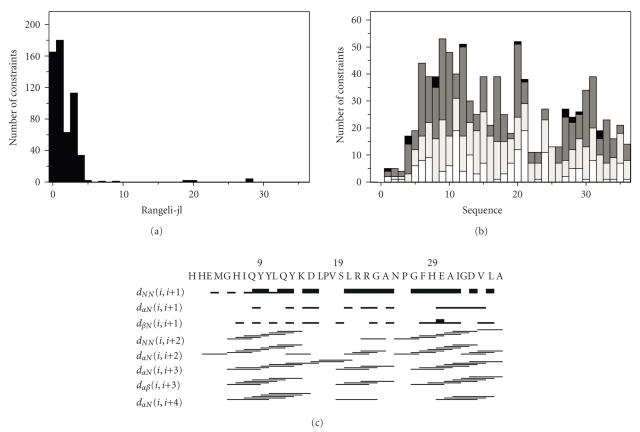
(a) Short-, medium-, and long-range connectivities. (b) Number of NOE constraints per residue (white, gray, dark gray, and black vertical bars represent, resp., intraresidue, sequential, medium-range and long-range connectivities). (c) Schematic representation of the sequential and medium range NOEs involving HN, H*α*, and H_*β*_ protons for Zn^2+^-ACE_N_(37) (corresponds to His^360^-Ala^396^ of the human somatic form).

**Figure 3:**
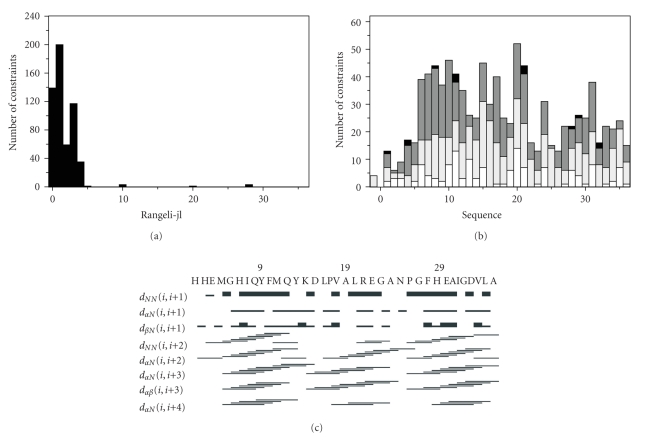
(a) Short-, medium-, and long-range connectivities. (b) Number of NOE constraints per residue (white, gray, dark gray, and black vertical bars represent, resp., intraresidue, sequential, medium-range, and long-range connectivities). (c) Schematic representation of the sequential and medium range NOEs involving HN, H*α*, and H_*β*_ protons for Zn^2+^-ACE_C_(37) (corresponds to His^958^-Ala^994^ of the human somatic form).

1318 and 1578 NOESY cross-peaks were assigned in both dimensions for ACE_C_(37), and ACE_N_(37), respectively, in TFE aqueous solution (TFE/H_2_0 2 : 1). The number of unique cross-peaks was 753 and 773 for ACE_C_(37) and ACE_N_(37), respectively. Their intensities were converted into upper limit distances through CALIBA [28]. The NOE-derived structural information extracted from the analysis of NOESY spectra acquired in aqueous TFE solutions under identical experimental conditions for both peptides were introduced to DYANA [29, 30] software for structure calculation (Figures 2 and 3). Structural calculations have been performed on IBM RISC6000 and xw4100/xw4200 HP Linux workstations. The family ensemble of Zn^2+^-ACE_N_(37) peptide presents root mean square deviation (RMSD) values of 0.65 ± 0.21 Å and 1.25 ± 0.24 Å for backbone and heavy atoms, respectively, and the average target function was found to be 0.39 ± 0.0164 Å^2^. The RMSD values of the Zn^2+^-ACE_C_(37) peptide were 0.55 ± 0.23 Å and 1.04 ± 0.27 Å for backbone and heavy atoms, respectively, and target function lies in the range 0.60  ± 4.78 × 10^−2^ Å^2^.

## 3. Results and Discussion

### 3.1. *α*-Helix Content Measurements through Circular Dichroism Data

**Figure 4:**
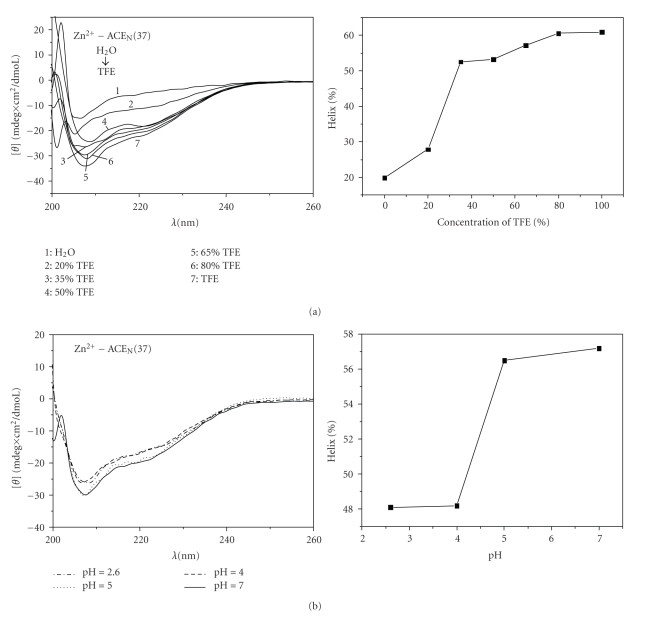
Circular dichroism spectra (left) and corresponding diagrams (right) of helical content through data analysis by CDNN software of (a) 2,2,2-trifluoroethanol (TFE) range from 0% to 100% of Zn^2+^-ACE_N_(37) samples, at pH = 5.0, *T* = 25°C, 50 mM Tris-HCl, and 200 mM NaCl and (b) of pH range from 2.6 to 7 of Zn^2+^-ACE_N_(37) samples, at 65% TFE, *T* = 25°C, 50 mM Tris-HCl, and 200 mM NaCl.

The Circular Dichroism (CD) experiments provided a qualitative determination of the peptide secondary structure elements in different TFE concentrations, pH values, and metal addition, leading to the determination of high resolution experimental conditions (Figure 4).

Specifically, the Zn^2+^-ACE_N_(37) peptide presents an unfolded structure with low *α*-helical content in aqueous solution. Low TFE concentrations (up to 20%) do not seem to have major effect on the peptide conformation (Figure 4(a)). However, the CD spectrum of the sample containing 35% TFE shows two intense minima at 208 and 220 nm, characteristics of *α*-helical structure. Thus, for TFE concentration ranging from 20% to 35%, an abrupt structural change takes place, leading to a dramatical increase of *α*-helix content from 26% to 52%. At higher TFE concentrations (50%–100%), no remarkable alteration of the helical content is noticed. In more detail, the *α*-helical content is increased from 54% to 60%, for TFE concentration increasing from 50% to 100%, indicating secondary structure stability of the Zn^2+^-ACE_N_(37) peptide at alcohol concentration greater than 50%. Similar results were obtained for the Zn^2+^-ACE_C_(37) peptide (data not shown). As a conclusion, the synthetic peptides exhibit a remarkable tendency to adopt helical conformation.

In order to investigate the pH effect on the *α*-helical content of the Zn^2+^-ACE_N_(37) peptide, CD measurements were performed in 65% of TFE at 25°C, at acidic, low acidic, and neutral pH values. Spectra representing two sets of pH values nearly overlap, thus suggesting that the secondary structure of ACE peptides exhibit minor differences at these pH values (Figure 4(b)). In particular, at acidic (pH 2.6) and mild acidic (pH 4.0) conditions, the Zn-containing ACE_N_(37) solutions possess approximately 48%  *α*-helical content while at pH values of 5.0 and 7.0, the helicity of the ACE_N_(37) in the presence of Zn^2+^ ions is found to be approximately 57.0%.

### 3.2. NMR Spectra Assignment of Zn^2+^-ACE_N_(37) and Zn^2+^-ACE_C_(37) Peptides

**Figure 5:**
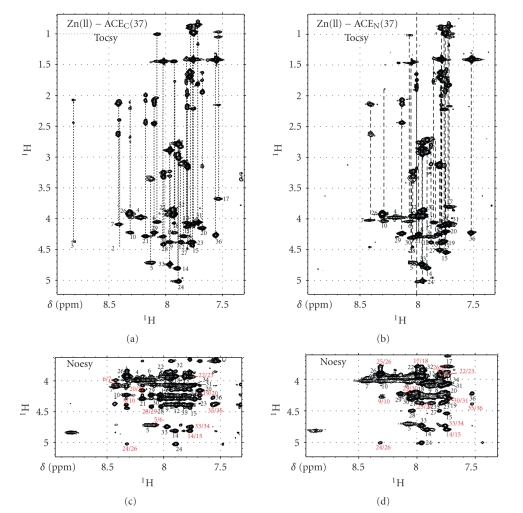
Fingerprint regions of 600 MHz TOCSY ((a) ACE_C_(37) and (b) ACE_N_(37)) and NOESY ((c) ACE_C_(37) and (d) ACE_N_(37)) spectra recorded at *T* = 298 K. The sequential connectivity pattern shown indicates the peptide sequence-specific resonance assignment.

Thirty-six out of 37 residues of the backbone of both Zn^2+^-ACE peptides have been identified through the analysis of the TOCSY spectra (Figure 5). ^1^H spin systems of the His, Phe and Tyr aromatic rings were identified with the combined use of [^1^H- ^1^H]- TOCSY and NOESY spectra (Tables 1 and 2). The two proline residues existing in each construct were found to be at *trans* conformation for both peptides manifested by strong H_*δ*_(i)Pro- H_*α*_(i-1) NOE connectivities.

### 3.3. NMR Solution Models of Zn^2+^-ACE_N_(37) & Zn^2+^-ACE_C_(37) Peptides

**Figure 6:**
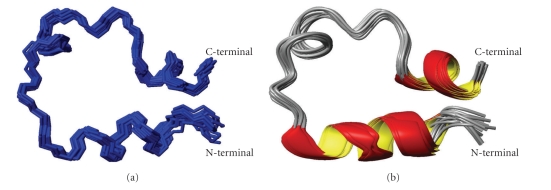
(a) Ensemble of DYANA 30 best models of the Zn^2+^-ACE_N_(37) (corresponds to His^360^-Ala^396^ of the human somatic form) calculated with NMR data. (b) Ribbon diagram of Zn^2+^-ACE_N_(37) peptide.

As far as the N-terminal Zn-binding motif of ACE_N_(37) peptide, which contains the two histidyl ligands, is concerned, no definite conformation could be determined due to conformational averaging. A 7-residue fragment close to the N-terminal (Gln^8^-Asp^15^) adopts helical structure, which consists partly of an *α*-helix for the 8-11 fragment and of a short 3_10_-helix for the rest of the four-residue segment. A second fragment comprised of 7 residues close to peptide C-terminal (His^29^- Leu^36^) adopts a well formed *α*-helical structure. As far as the intermediate fragment of the 23-residue spacer between the two binding motifs is concerned, no helical conformation has been identified. The proximity of the two “active sites helices” is manifested by long-range NOEs concerning backbone and side-chain protons of His^2^–Glu^30^, Gly^5^–Gly^34^, Tyr^9^–Phe^28^ see (Figure  S1) in Supplementary Material available online at doi: 10.1155/2010/820476, as well as Tyr^9^–His^30^ (Figure 6).

**Figure 7:**
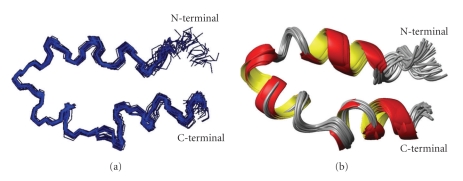
(a) Ensemble of DYANA 30 best models of the Zn^2+^-ACE_C_(37) (corresponds to His^958^- Ala^994^of the human somatic form), calculated with NMR data. (b) Ribbon diagram of Zn^2+^-ACE_C_(37) peptide.

The Zn^2+^-ACE_C_(37) backbone is characterized by the high content of helical structure. Two helical conformations were observed at both N- and C-termini, spanning residues His^6^–Lys^14^ and Phe^28^–Val^35^, respectively. Moreover, a 3_10_- helix comprised of a 5-residue segment (Ala^19^-Gly^23^) has been identified for the intermediate fragment. In accordance with the Zn^2+^-ACE_N_(37) peptide, the two zinc-binding motifs of Zn^2+^-ACE_C_(37) are in spatial proximity as manifested by long-range NOEs, such as those between His^6^/Ile^7^ and His^29^ as well as Gln^8^/Gln^12^ with Ala^19^ (Figure 7).

### 3.4. Solution Structure of Zn^2+^-ACE_N_(37) versus Zn^2+^-ACE_C_(37)

**Figure 8:**
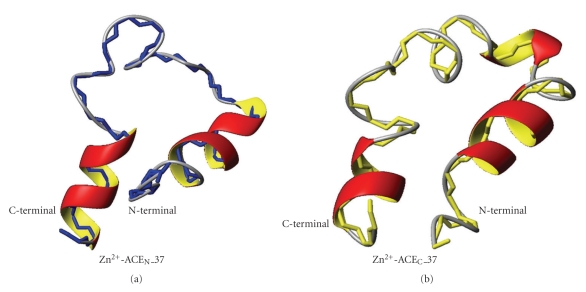
Backbone and ribbon representation of the solution structures of both Zn^2+^-ACE_N_(37) (a) (corresponds to His^360^-Ala^396^ of the human somatic form) and Zn^2+^-ACE_C_(37) (b) (corresponds to His^958^- Ala^994^of the human somatic form).

Although the overall fold of the two Zn^2+^-ACE peptides exhibits significant similarities, some striking differences, mainly related to the helical extent are detected (Figure 8). The double substitution of Tyr^10^ and Leu^11^ in ACE_N_(37) with Phe^10^ and Met^11^ in ACE_C_(37) does not impose any structural change, and the *α*-helix conformation of this segment remains. The nonhelical character of the N-terminal pentapeptide, which comprises the first Zn^2+^-binding motif, followed by a helical domain of eight to nine residues is conserved in both ACE peptides. However, small differences in NOEs are observed, regarding residues Ile^7^, Phe^10^/Tyr^10^, and Leu^11^/Met^11^ in ACE_N_(37) and ACE_C_(37) peptide, respectively. Because of the differentiation in position 10, the long range NOEs of the vicinal Tyr^9^ with Phe^28^ and His^29^ that are presented in ACE_N_(37) peptide, are not detected in ACE_C_(37). Furthermore, a long-range NOE between H_*ε*_ of Arg^21^ and H_*ε*_ of Phe^28^ detected only in Zn^2+^-ACE_N_(37) peptide suggests the existence of a loop with the two residues coming close to each other, confirming a tertiary slight structural difference among the two peptides in terms of the orientation of the two zinc-binding motif helices. In ACE_C_(37) peptide, the two side chains of these residues are oriented almost parallel to each other pointing though to opposite orientation providing a more “extended” conformation for this segment. Additionally, a helix of the C-termini is observed in both peptides. 

The substitutions of the amino acids at positions 19 (Ser^19^/Ala^19^) and 22 (Arg^22^/Glu^22^) of the peptide sequence seem to differentiate the structure of the two peptides. The most important diversity among the structures of the ACE_N/C_ peptides regards the 5-residue spanning *α*-helix (Ala^19^-Gly^23^) of the intermediate spacer between the two zinc-binding motifs, detected only in the ACE_C_(37) peptide. The absence of the intermediate helix in the ACE_N_(37) peptide probably provides a less constrained domain to the N-active site of the somatic form in a crucial socket region for the substrate binding. As a conclusion, the presence or absence of the helical structure of the intermediate spacer determines the relative position of the two terminal helices and differentiates the active-site cavity structure and volume, where the substrate is accommodated. Both active sites of the somatic form act as carboxy dipeptidase, hydrolyzing the amide bond and releasing the C-terminal dipeptide from a native substrate [1]. Additionally, only the ACE_N_ domain presents endopeptidase activity, by releasing the C-terminal tripeptide of the GnRH hormone and the tetrapeptide of the native octapeptide Enkephalin [12]. Accommodation of these peptides in active or not mode for proteolytic cleavage, might be influenced by the conformation of the interhelical spacer.

### 3.5. NMR Solution Structure versus X-Ray

**Figure 9:**
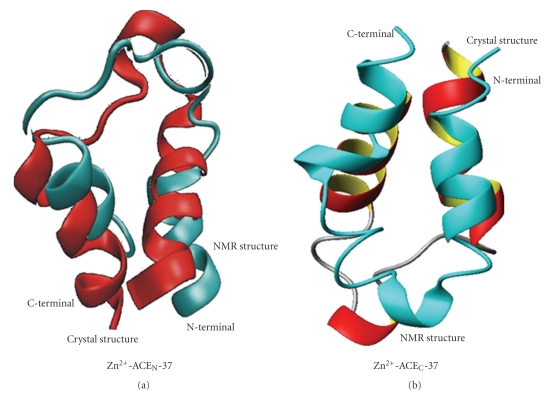
Superimposition of the crystal structure (in red) and the solution structure derived from NMR data (in cyan) of Zn^2+^-ACE_N_(37) peptide (a) and Zn^2+^-ACE_C_(37) peptide (b).

The overall fold of both synthetic peptides solution structures presents high similarity to the corresponding domain of the crystal structure of the testis ACE isoform [19] (tACE has identical sequence with the C-domain of the ACE somatic isoform) and the somatic ACE_N_ domain [18]. Minor differences exist regarding the length of the helices at the termini of the peptides (Figure 9). The backbone RMSD value for the family of 20 NMR structures of the ACE_N_ peptide and the crystal structure (2C6F) was found to be 1.42 Å and the corresponding RMSD value calculated for the mean NMR structure and the X-ray structure was found to be 3.802 Å. The backbone RMSD value for the family of 20 NMR structures of the ACE_C_ peptide and the crystal structure (1O8A) was found to be 0.775 Å and the corresponding RMSD value calculated for the mean NMR structure and the X-ray structure was found to be 3.595 Å.

In the tACE X-ray structure, two helical fragments are present at the N- and C- terminal, both comprising a 12-residue segment (Figure 9). For ACE_N_ crystal structure the N-terminal 13-residue fragment exhibits a helical conformation, as well as the entire C-terminal 12-residue fragment (Figure 9(a)). Regarding the intermediate spacer among the two zinc-binding motifs, an additional helix region consisting of 3 amino acids is present at both tACE and ACE_N_ X-ray structures. In the case of the NMR derived structure, a shorter helical fragment has been observed for both termini. The obtained data for the Zn^2+^-ACE_N_(37) suggest that as far as the N-terminal is concerned a 10-residue fragment (His^6^-Asp^15^) and as far as the C-terminal is concerned a 8-residue fragment (His^29^-Leu^36^) exhibit helical conformation. Similar results have been obtained for the synthetic peptide representing the C-catalytic domain of the human somatic form and the corresponding domain of the testis form. Differences among the ACE catalytic site maquettes and X-ray structures might be due to the fact that the native N- and C-domains exhibit compact structures, and packing of the structure elements in the interior of the enzyme where the catalytic center is cited diminishes the conformational flexibility of the two active-site helices.

Concerning the intermediate spacer between the two zinc-binging motifs, the X-ray models present an additive 3-residue helical segment residue, concerning residues 18-20 (numbering of crystal structure: tACE: Val^399^-Leu^401^; ACE_N_: Val^377^-Leu^379^). NMR solution structure of the Zn^2+^-ACE_C_(37) peptide resulted in significantly similar conformation. At ACE_C_(37) peptide, a 3_10_ helix is formed for the 5-residue segment Ala^19^-Gly^23^. Instead, no helical conformation has been detected for the Zn^2+^-ACE_N_(37) peptide, according to the NMR data and the DYANA calculations. However, both structures of tACE and sACE_N_ have been further solved in complex with the typical ACE inhibitor, lisinopril [19] (Figures  S2 and S3). Among them, a helical structure is identified for the intermediate spacer only in the tACE-lisinopril structure, while in the sACE_N_-lisinopril model the helical segment is absent. The modifications of positions 19 and 22 in the sACE_N_ peptide are probably playing an important role in the structural diversity of the spacer and are consequently crucial for the different activity and substrate specificity of these two active sites.

As a conclusion, the conformation of the synthetic peptides and the orientation of the two helical motifs upon zinc coordination are remarkably similar to the native structure, indicating the ACE catalytic site maquettes as reliable models of the enzyme active centre. The detected differences are clearly depended on the physicochemical properties of the peptides in solution compared to the crystal structures.

## 4. Conclusions

NMR studies of the synthetic peptides generated structures that successfully simulate the crystal structures of ACE C- and N-domains. Circular dichroism experiments provided important data, compensate not only for experimental conditions of the NMR analysis, but also for the elucidation of the structural characteristics of the two peptides, corresponding to the two somatic isoform ACE catalytic sites domains. The TFE use in aqueous mixtures, the proper pH value, as well as the presence of the Zn-ion create a solution environment, in which peptides adopt a similar fold to the native structure. The NMR data and the computational analysis led to structural models of the peptides, which are in great agreement with the X-ray structures. The secondary structure features of the peptides that correspond to the sequence of the ACE catalytic sites X-ray structures Zn^2+^-ACE_N_(37) to ACE_N_; Zn^2+^-ACE_C_(37) to tACE present minor differences compared to the crystal structures. Furthermore, the ACE maquettes and the X-ray structures present significant similarities in the orientation of the active site helices, in respect of the position of the zinc ligands for metal coordination. On the other hand, the peptide representing the N-catalytic site lacks the helix of the intermediate region. Thus, the two terminal helices of the C-catalytic site maquette are found closer than those in ACE_N_, illustrating potential differences into the catalytic site pocket for substrate selectivity, binding, and accommodation. This crucial difference might possibly explain the functional diversity of the two somatic isoforms of the human ACE. This approach might be helpful in the reconstitution of other enzymes' active sites with unknown structures. In *gluzincins*, the spacer among the two zinc-binding motifs is regarded as of great importance for the specificity of the native substrates and external ligands, such as inhibitors. As far as somatic ACE is concerned, both active sites exhibit carboxy dipeptidase function while only the N-catalytic site exhibits also endopeptidase activity.

In the work reported here, crucial structural data for solution conformations have been extracted even though the crystal structure of testis ACE has been solved. Due to the functional diversity of the two ACE active sites, the “structure-based drug design” of the next generation of pharmaceutical agents, which specifically inhibit one of the two zinc catalytic domains of somatic ACE, is of major importance for the preferential modulation of ACE proteolytic activity towards a more effective treatment of hypertensive patients.

## Supplementary Material

The *φ* and *ψ* values of the dihedral angles of both peptides are provided in the Supplementary Material (Tables S1 and S2). Moreover, characteristic long range NOEs for ACEN(37) peptide are displayed in figure S1 of the S.I. Figures S2 and S3 depict the superimposition among the X-ray structures and the solution structures of the studied peptides.

## Figures and Tables

**Table 1:** ^1^H, ^15^N, and ^13^C chemical shifts (ppm) of the residues in the Zn^2+^-ACE_N_(37) peptide at 298K (H2O/TFE-*d2* 34%/66% v/v. pH = 4.9).

Residue		HN/**N**	H*α*/**C** **α**	H*β*/**C** **β**	Other
1	His		4.45		H*δ* *2* 7.30; H*ε* *1* 8.44
			**55.39**		**C** **δ** 120.02 **C** **ε** **1** 139.44
2	His	7.99	4.84	3.38/3.29	H**δ**2 7.32; H**ε**1 8.45
		**N **117.01	**55.84**	**28.45**	**C** *δ*2 120.05 **C** *ε*1 137.64
3	Glu	8.45	4.47	2.15/2.14	*γ*CH_3_ 2.65/2.62
		**N **121.15	**56.81**	**31.91**	**C** **γ** 31.90
4	Met		4.35	2.13/2.09	H*γ* 2.49/2.44
			**57.51**	**33.70**	**C** *γ* 34.78
5	Gly	8.22	3.98		
		**N **109.59	**45.79**		
6	His	8.22	4.72	3.41/3.39	H*δ* *2* 7.26; H*ε* *1* 8.50
		**N**. 119.31	**57.08**	**28.57**	**C** **δ** 119.93 **C** **ε** **1 **136.27
7	Ile	8.11	4.05	2.07	H*γ* 1.70/1.32; *γ*CH_3_ 1.03 *δ*CH_3_ 0.98
		**N **122.73	**64.30**	**38.04**	**C** **γ** **1 ** 28.30** C** **γ** **2 **16.79** C** **δ** 12.22
8	Gln	8.47	4.03	2.14/2.11	H*γ* 2.44; *δ*NH_2_ 6.55/7.14
		**N **120.70	**59.13**	**28.91**	**C** **γ** 33.65 **N** **ε** **2 **110.97
9	Tyr	7.84	4.26	3.12	H*δ* 6.96; H*ε* 6.75;
		**N **120.77	**60.88**	**38.03**	**C** **δ** **1 **133.66 **C** **ε** **1 **117.77
10	Tyr	7.87	4.29	3.26/3.22	H*δ* 7.19; H*ε* 6.89;
		**N **120.23.	**60.82**	**38.11**	**C** **δ** **1 **132.73 **C** **ε** **1 **117.87

11	Leu	8.34	4.04	1.99/1.91	H*γ* 1.53; *δ*CH_3_ 0.94
		**N **119.99	**57.26**	**41.69**	**C** **γ** 28.36** C** **δ** 21.77/24.48
12	Gln	7.81	4.11	2.01/1.91	H*γ* 2.24/2.16; *δ*NH_2_ 6.42/6.99
		**N **118.28	**57.58**	**28.81**	**C** **γ** 33.54 **N** **ε** **2 ** 110.97
13	Tyr	7.91	4.38	3.10/2.82	H*δ* 7.00; H*ε* 6.76
		**N **120.43	**59.51**	**35.59**	**C*δ*1 **132.82 **C** **ε** **1 **117.83
14	Lys	7.90	4.06	1.82/1.79	H*γ* 1.39; *δ*CH_3_ 1.63; *ε*CH_3_ 3.03
		**N **120.21	**57.55**	**31.61**	**C** **γ** 23.71 **C** **δ** 28.57 **C** **ε** 42.06
15	Asp	7.93	4.80	2.92/2.76
		**N** 118.92	**53.56**	**39.48**
16	Leu	7.76	4.54	1.79	H*γ*1.58; *δ*CH_3_ 0.94/0.94
		**123.89**	**54.95**	**41.64**	**C** **γ** 28.43** C** **δ** 23.05/24.36
17	Pro		4.45	2.39/1.95	H*γ* 2.02/1.97; H*δ* 3.88/3.64
			**63.27**	**31.32**	**C** **γ** 26.69** C** **δ** 50.27
18	Val	7.74	3.79	2.17	*γ*CH_3_ 1.06/1.01
		**N **120.40	**65.07**	**31.81**	**C** **γ** 20.89/20.12
19	Ser	8.02	4.23	3.99/3.95
		**N **116.85	**60.48**	
20	Leu	7.82	4.36	1.77	H*γ* 1.68; *δ*CH_3_ 0.94/0.89
		**N **122.93	**56.20**	**41.96**	**C** **γ** 28.50 **C** **δ** 22.29/24.73

21	Arg	7.77	4.20	1.93	H*γ* 1.76/1.65; H*δ* 3.20; H*ε* 7.05
		**N **120.34	**57.49**	**30.15**	**C** **γ** 26.99** C** **δ** 43.13** N*ε***135.56
22	Arg	8.05	4.28	1.89/1.85	H*γ* 1.75/1.64; H*δ* 3.17; H*ε* 7.15
		**N **121.00	**57.05**	**30.14**	**C*γ***26.92** C** **δ** 43.07** N** **ε** 135.62
23	Gly	8.00	3.95
		**N **109.07	**45.43**
24	Ala	7.81	4.39	1.43
		**N **124.37	**52.21**	**19.05**
25	Asn	7.98	5.02	2.96/2.79	*δ*NH_2_ 6.63/7.47
		**N **119.26	**51.13**	**39.17**	**N** **δ** **2 **113.31
26	Pro		4.44	2.32/1.98	H*γ* 2.07/2.00; H*δ* 3.92/3.83
			**61.79**	**29.10**	**C** **γ** 26.76 **C** **δ** 50.28
27	Gly	8.34	3.94
		**N ** 108.58	**43.10**
28	Phe	7.83	4.50	3.15/3.13	H*δ* 7.25; H*ε* 7.29; H*ζ* 7.18
		**N **122.56	**56.50**	**36.59**	**C** **δ** 130.90/129.60 **C** **ε** 130.95/129.53 **C** **ζ** 131.21
29	His	8.05	4.44	3.32/3.25	H*δ* *2* 7.31; H*ε* *1* 8.51
		**N **119.37	**54.41**	**25.86**	**C** **δ** **2 **119.91 **C** **ε** **1 ** 139.03
30	Glu	8.15	4.23	2.15/2.10	*γ*CH_3_ 2.45
		**N **121.57	**54.73**	**24.95**	**C** **γ** 33.54

31	Ala	8.07	4.31	1.46	
		**N **125.19	**51.07**	**15.87**	
32	Ile	7.76	4.06	1.83	H*γ* 1.44/1.18; *γ*CH_3_ 0.88; *δ*CH_3_ 0.82
		**N **118.79	**61.44**	**35.60**	**C** **γ** **1 ** 27.34** C** **γ** **2 ** 16.84** C** **δ** 12.08
33	Gly	7.96	3.95/3.87
		**N **110.69	**43.26**
34	Asp	7.97	4.74	2.89
		**N **120.70	**51.72**	**36.82**
35	Val	7.78	4.08	2.23	*γ*CH_3_ 1.00
		**N **120.34	**60.78**	**29.97**	**C** **γ** 20.31/24.50
36	Leu	7.82	4.40	1.72	H*γ* 1.38; *δ*CH_3_ 0.92/0.88
		**N **122.65	**52.58**	**39.65**	**C** **γ** 27.26 **C** **δ** 24.16/22.01
37	Ala	7.56	4.25	1.43
		**N **128.06	**50.34**	**16.51**

**Table 2:** ^1^H, ^15^N, and ^13^C chemical shifts (ppm) of the residues in the Zn^2+^-ACE_C_(37) peptide at 298K (H_2_O/TFE-*d2* 34%/66% v/v. pH = 4.9).

Residue		HN/**N**	H*α*/**C** **α**	H*β*/**C** **β**	Other
1	His		4.44		H*δ* *2* 7.34; H*ε* *1* 8.48
			**55.45**		**C** **δ** **2** 120.14 **C** **ε** **1** 141.76
2	His	7.92	4.83	3.41/3.37	H*δ* *2* 7.31; H*ε* *1* 8.42
		**N** 117.71	**55.86**	**28.53**	** C** **δ** **2 **119.85** C** **ε** **1 **136.29
3	Glu	8.44	4.47	2.15	*γ*CH_3_ 2.64
		**N **121.00	**56.93**	**31.82**	**C** **γ** 31.95
4	Met		4.30	2.12/2.06	H*γ* 2.44;
			**58.52**	**33.59**	**C*γ***34.85
5	Gly	8.22	3.98
		**N ** 109.22	**45.83**
6	His	8.22	4.69	3.39	H*δ* *2* 7.25; H*ε* *1* 8.50
		**N**.119.42	**57.33**	**28.64**	**C** **δ** **2 **119.79 **C** **ε** **1 **138.23
7	Ile	8.06	4.04	2.07	H*γ* 1.71/1.31; *γ*CH_3_ 1.03 *δ*CH_3_ 0.97
		**N **122.46	**64.13**	**38.09**	**C** **γ** **1 ** 28.41** C** **γ** **2 **16.81** C** **δ** 12.18
8	Gln	8.44	4.08	2.15/2.11	H*γ* 2.42; *δ*NH_2_ 6.54/7.13
		**N **121.01	**59.04**	**28.57**	**C** **γ** 33.52 **N** **ε** **2 **110.83
9	Tyr	7.90	4.27	3.12	H*δ* 6.95; H*ε* 6.76
		**N **120.96	**61.02**	**38.21**	**C** **δ** **1 **132.67 **C** **ε** **1 **117.74
10	Phe	8.07	4.37	3.33/3.26	H*δ* 7.31; H*ε* 7.37 H*ζ* 7.34
		**N **120.42	**60.74**	**38.84**	**C** **δ** 131.03/129.50 **C** **ε** 131.00/129.43 **C** **ζ** 131.36

11	Met	8.43	4.21	2.23/2.15	H*γ* 2.79/2.72
		**N **118.65	**57.43**	**33.58**	**C** **γ** 32.35
12	Gln	7.81	4.14	2.00/1.90	H*γ* 2.22/2.17; *δ*NH_2_ 6.40/6.70
		**N **118.68	**57.74**	**28.75**	**C** **γ** 33.50 **N** **ε** **2 ** 110.86
13	Tyr	7.92	4.38	3.13/2.82	H*δ* 7.00; H*ε* 6.74
		**N **120.02	**59.46**	**39.33**	**C** **δ** 132.81 **C** **ε** 117.84
14	Lys	7.85	4.05	1.78/1.81	H*γ* 1.39; *δ*CH_3_ 1.64; *ε*CH_3_ 3.05
		**N **120.09	**57.76**	**31.58**	**C** **γ** 23.68 **C** **δ** 28.76 **C** **ε** 42.19
15	Asp	7.90	4.83	2.93/2.77
		**N **118.79	**53.53**	**39.51**
16	Leu	7.78	4.46	1.79	H*γ* 1.77; *δ*CH_3_ 0.943/0.97
		**123.98**	**56.77**	**41.25**	**C** **γ** 26.83** C** **δ** 23.25/24.05
17	Pro		4.39	2.41/1.89	H*γ* 2.11/2.07; H*δ* 3.66/3.87
			**64.61**	**31.21**	**C** **γ** 26.79** C** **δ** 50.16
18	Val	7.60	3.68	2.17	*γ*CH_3_ 1.07/0.98
		**N **120.40	**65.47**	**31.77**	**C** **γ** 20.23/21.25
19	Ala	7.94	4.08	1.46
		**N **123.38	**54.68**	**18.08**
20	Leu	7.95	4.25	1.78	H*γ* 1.63; *δ*CH_3_ 0.92/0.89
		**N **123.48	**56.69**	**41.91**	**C** **γ** 27.43 **C** **δ** 22.06/24.33

21	Arg	7.70	4.15	1.96	H*γ* 1.75/1.63; H*δ* 3.15; H*ε* 7.06
		**N **120.14	**57.82**	**30.09**	** C** **γ** 26.80** C** **δ** 42.96** N** **ε** 135.38
22	Glu	8.23	4.30	2.10/2.00	*γ*CH_3_ 2.46/2.42
		**N **119.39	**57.01**	**28.25**	**C** **γ** 33.01
23	Gly	7.95	3.93
		**N **108.22.	**45.70**
24	Ala	7.78	4.40	1.45
		**N **123.97	**52.25**	**18.47**
25	Asn	7.90	5.03	3.03/2.81	*δ*NH_2_ 6.61/7.50
		**N **118.78	**51.01**	**39.09**	**N** **δ** **2 **113.04
26	Pro		4.45	2.35/2.01	H*γ* 2.07/2.02; H*δ* 3.91/3.85
			**64.33**	**31.50**	**C** **γ** 26.84 **C** **δ** 50.43
27	Gly	8.34	3.94		
		**N** 108.27	**45.73**		
28	Phe	7.83	4.49	3.16	H*δ* 7.24; H*ε* 7.28; H*ζ* 7.15
		**N **122.54	**59.19**	**39.20**	**C** **δ** 130.90/129.43 **C** **ε** 130.94/129.55 **C** **ζ** 131.15
29	His	8.03	4.40	3.32/3.28	H*δ* *2* 7.33; H*ε* *1* 8.51
		**N **118.86	**57.20**	**28.38**	**C** **δ** **2 **119.94 **C** **ε** **1 ** 135.99
30	Glu	8.15	4.22	2.16/2.12	*γ*CH_3_ 2.46
		**N **121.32	**57.33**	**27.91**	**C** **γ** 33.66

31	Ala	8.05	4.28	1.47
		**N **124.86	**53.62**	**18.02**
32	Ile	7.75	4.05	1.89	H*γ* 1.41/1.18; *γ*CH_3_ 0.85; *δ*CH_3_ 0.80
		**N **118.32	**64.06**	**38.03**	**C** **γ** **1 ** 27.29** C** **γ** **2 ** 16.80** C** **δ** 12.02
33	Gly	7.94	3.90/3.86
		**N **110.35	**45.81**
34	Asp	7.97	4.74	2.88
		**N **120.63	**54.30**	**39.45**
35	Val	7.77	4.08	2.24	*γ*CH_3_ 0.99
		**N **120.04	**63.48**	**32.45**	**C** **γ** 20.23/23.01
36	Leu	7.83	4.39	1.71	H*γ* 1.63; *δ*CH_3_ 0.92/0.88
		**N **125.55	**55.14**	**42.14**	**C** **γ** 28.74 **C** **δ** 24.36/21.98
37	Ala	7.55	4.24	1.43
		**N **128.05	**52.89**	**19.23**

## Abbreviations

ACE: Angiotensin-I Converting Enzyme
sACE: ACE somatic isoform
tACE: ACE testis isoform
ACE_N_: N-domain of sACE
ACE_C_: C-domain of sACE
TFE: 2,2,2-trifluoroethanol
DQF-COSY: Double-quantum-filtered phase-sensitive correlated spectroscopy
Tris: Tris(hydroxymethyl)aminomethane
TOCSY: Total Correlated Spectroscopy
NOE: Nuclear Overhauser effect
NOESY: Nuclear Overhauser effect spectroscopy
TPPI: Time-proportional phase incrementation
WATERGATE: Water suppression by gradient-tailored excitation
RMSD: Root Mean Square Deviation
REM: Restrained Energy Minimization
GPCRs: G-protein coupled receptors.
