# Supplementary material for: Synthetic Peptides as Structural Maquettes of Angiotensin-I Converting Enzyme Catalytic Sites
Source: Bioinorg Chem Appl. 2010 Jun 9;2010:820476. doi: 10.1155/2010/820476 (PMC2902127; doi:10.1155/2010/820476)
Supplement: Supplementary file 1 — The φ and ψ values of the dihedral angles of both peptides are provided in the Supplementary Material (Tables S1 and S2). Moreover, characteristic long range NOEs for ACEN(37) peptide are displayed in figure S1 of the S.I. Figures S2 and S3 depict the superimposition among the X-ray structures and the solution structures of the studied peptides. [file 820476.f1.pdf]

Synthetic Peptides as Structural Maquettes of Angiotensin-I Converting Enzyme Catalytic Sites

Zinovia Spyranti[1a], Athanassios S. Galanis[1a], George Pairas[1], Georgios A. Spyroulias[1*], Evy Manessi-Zoupa[2] and Paul Cordopatis[1*]

SUPPORTING INFORMATION

Table S1: φ and ψ dihedral angles of the Zn^{2+}-ACE_C(37) peptide at 298K (H_2O/TFE-*d2* 34%/66% v/v. pH=4.9).

Table S2: φ and ψ dihedral angles of the the Zn^{2+}-ACE_N(37) peptide at 298K (H_2O/TFE-*d2* 34%/66% v/v. pH=4.9).

Figure S1: Fingerprint regions of 600- MHz NOESY of ACE_N(37) peptide recorded at T= 298 K. Characteristic long-range NOE connectivities between Tyr 368 with His 388, as well as with Phe 387 present only in the ACEN_37 peptide.

Figure S2: **Left:** Superimposition of Zn^{2+}-ACE_C(37) DYANA 20 best models calculated with NMR data in blue and the crystal structure of ACE_C in complex with lisinopril in yellow (pdb code:***1O86***). The calculated RMSD value is 0.775 for the backbone atoms. **Right:** Ribbon representation of the crystal structure of ACE_C in complex with lisinopril and the mean NMR calculated structure of Zn^{2+}-ACE_C(37). The calculated RMSD value is 3.595 for the backbone atoms.

Figure S3: Left: Superimposition of Zn^{2+}-ACE_N(37) DYANA 20 best models calculated with NMR data in blue and the crystal structure of ACE_C in complex with lisinopril in yellow (pdb code:***2C6N***). The calculated RMSD value is 1.42 for the backbone atoms. **Right:** Ribbon representation of the crystal structure of ACE_N in complex with lisinopril and the mean NMR calculated structure of Zn^{2+}-ACE_N(37). The calculated RMSD value is 3.802 for the backbone atoms.

Table S1: φ and ψ dihedral angles (in degrees) of the Zn^{2+}-ACE_C(37) peptide at 298K (H_2O/TFE-*d2* 34%/66% v/v. pH=4.9).

Residue		φ	ψ
1	His	---	**-60.2**+/-77.2
2	His	**-28.7+/-**71.6	**133.7**+/-81.4
3	Glu	**-117.3**+/-94.6	**-20.5**+/-10.0
4	Met	**-123.9+/-**20.0	**28.5**+/-13.6
5	Gly	**-130.3**+/-25.8	**-79.5+/-**16.0
6	His	**-50.9**+/-6.6	**-34.2+/-**4.7
7	Ile	**-65.0**+/-1.3	**-37.4+/-**2.6
8	Gln	**-50.1**+/-2.6	**-40.6+/-**1.8
9	Tyr	**-69.7**+/-2.3	**-45.9+/-**1.0
10	Phe	**-62.8**+/-1.0	**-30.3+/-**2.1
11	Met	**-84.3**+/-2.9	**-26.3**+/-2.2
12	Gln	**-68.8**+/-2.2	**-30.0**+/-1.4
13	Tyr	**-50.6**+/-1.1	**-36.2**+/-3.0
14	Lys	**-74.6**+/-1.0	**-25.8**+/-0.8
15	Asp	**-87.2**+/-2.3	**54.5**+/-1.4
16	Leu	**-64.6**+/-1.1	**-42.8**+/-0.3
17	Pro	---	**-1.8**+/-1.2
18	Val	**-126.4**+/-1.4	**1.4**+/-0.7
19	Ala	**-60.1**+/-1.4	**-15.5**+/-0.9
20	Leu	**-85.6**+/-0.9	**5.8**+/-1.7
21	Arg	**-135.4**+/-2.7	**-43.5**+/-0.4
22	Glu	**-83.5**+/-5.5	**-41.3**+/-2.2
23	Gly	**-79.3**+/-1.7	**-10.3**+/-5.3
24	Ala	**-102.0**+/-10.2	**14.8**+/-4.0
25	Asn	**-122.2**+/-5.8	**75.1**+/-7.8
26	Pro	---	**-10.6**+/-2.7
27	Gly	**-112.9**+/-9.2	**20.0**+/-3.9
28	Phe	**-138.0**+/-2.8	**-44.8**+/-3.3
29	His	**-65.9**+/-4.4	**-23.1**+/-1.8
30	Glu	**-112.8**+/-1.3	**-15.9**+/-1.7
31	Ala	**-77.0**+/-3.3	**-54.7**+/-1.3
32	Ile	**-59.6**+/-1.1	**-18.2**+/-1.4
33	Gly	**-104.3**+/-1.8	**-17.3+/-**1.2
34	Asp	**-86.4**+/-3.9	**-31.9**+/-3.3
35	Val	**-63.3**+/-3.9	**-49.4**+/-1.9
36	Leu	**-64.6**+/-8.7	**-11.0**+/-6.4
37	Ala	**-149.3**+/-14.5	**-60.3**+/-53.3

Table S2: φ and ψ dihedral angles (in degrees) of the the Zn^{2+}-ACE_N(37) peptide at 298K (H_2O/TFE-*d2* 34%/66% v/v. pH=4.9).

Residue		φ	ψ
**1**	**His**	**---**	**80.1**+/-79.0
**2**	**His**	**-116.1**+/-80.7	**98.4**+/-82.1
**3**	**Glu**	**-75.2+/-**77.2	**35.5**+/-17.3
**4**	**Met**	**-59.3**+/-81.1	**25.7**+/-61.3
**5**	**Gly**	**128.3+/-**38.9	**7.0**+/-31.6
**6**	**His**	**-89.1+/-**19.8	**-43.9**+/-12.3
**7**	**Ile**	**-61.4+/-**5.6	**-45.2**+/-7.6
**8**	**Gln**	**-63.2+/-**6.1	**-28.0**+/-4.0
**9**	**Tyr**	**-73.0+/-**3.9	**-17.1**+/-4.3
**10**	**Tyr**	**-112.1+/-**3.0	**-42.4**+/-4.5
**11**	**Leu**	**-52.9+/-**6.1	**-45.2**+/-1.6
**12**	**Gln**	**-78.0**+/-1.8	**-22.0**+/-2.5
**13**	**Tyr**	**-84.0**+/-4.5	**-1.0**+/-4.3
**14**	**Lys**	**-41.7**+/-8.6	**-39.6**+/-9.2
**15**	**Asp**	**-165.3+/-**10.2	**86.1**+/-30.4
**16**	**Leu**	**-107.2+/-**15.4	**160.5**+/-1.0
**17**	**Pro**	**---**	**-6.2**+/-2.2
**18**	**Val**	**37.9**+/-1.9	**87.2**+/-2.9
**19**	**Ser**	**-129.1+/-**8.2	**-44.1**+/-3.4
**20**	**Leu**	**-96.4**+/-4.3	**6.6**+/-6.8
**21**	**Arg**	**-124.2**+/-7.8	**-44.2**+/-4.1
**22**	**Arg**	**-42.5**+/-8.5	**-27.4**+/-6.7
**23**	**Gly**	**-140.4+/-**19.3	**-13.7**+/-37.6
**24**	**Ala**	**-103.6**+/-35.2	**-87.4**+/-6.6
**25**	**Asn**	**43.2**+/-31.9	**67.1**+/-11.8
**26**	**Pro**	**---**	**-9.5**+/-4.9
**27**	**Gly**	**142.1**+/-9.4	**101.4**+/-8.6
**28**	**Phe**	**-128.7+/-**14.7	**-44.2**+/-5.6
**29**	**His**	**-47.1**+/-9.4	**-57.0**+/-10.3
**30**	**Glu**	**-83.6**+/-2.2	**-24.0**+/-1.4
**31**	**Ala**	**-58.0**+/-6.9	**-52.5**+/-3.4
**32**	**Ile**	**-58.3**+/-2.9	**-42.4**+/-0.4
**33**	**Gly**	**-65.9**+/-2.3	**-33.0**+/-1.1
**34**	**Asp**	**-72.9**+/-3.0	**-9.8**+/-2.2
**35**	**Val**	**-96.8+/-**4.6	**-26.9**+/-4.1
**36**	**Leu**	**-104.1**+/-25.4	**6.3**+/-53.4
**37**	**Ala**	**-126.7**+/-59.8	**-93.9**+/-77.1

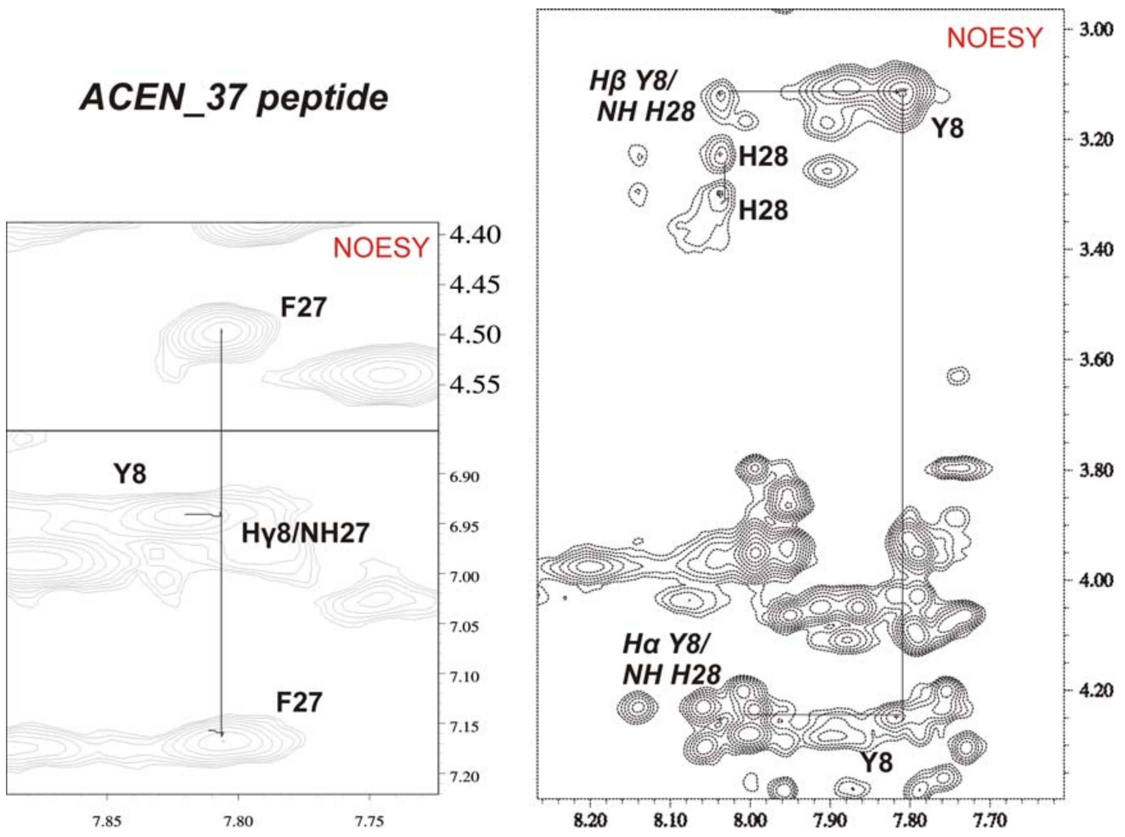

Figure S1: Fingerprint regions of 600- MHz NOESY of Zn^{2+}-ACE_N(37) peptide recorded at T= 298 K. Characteristic long-range NOE connectivities between Tyr^8 with His^{28}, as well as with Phe^{27} present only in the Zn^{2+}-ACE_N(37) peptide.

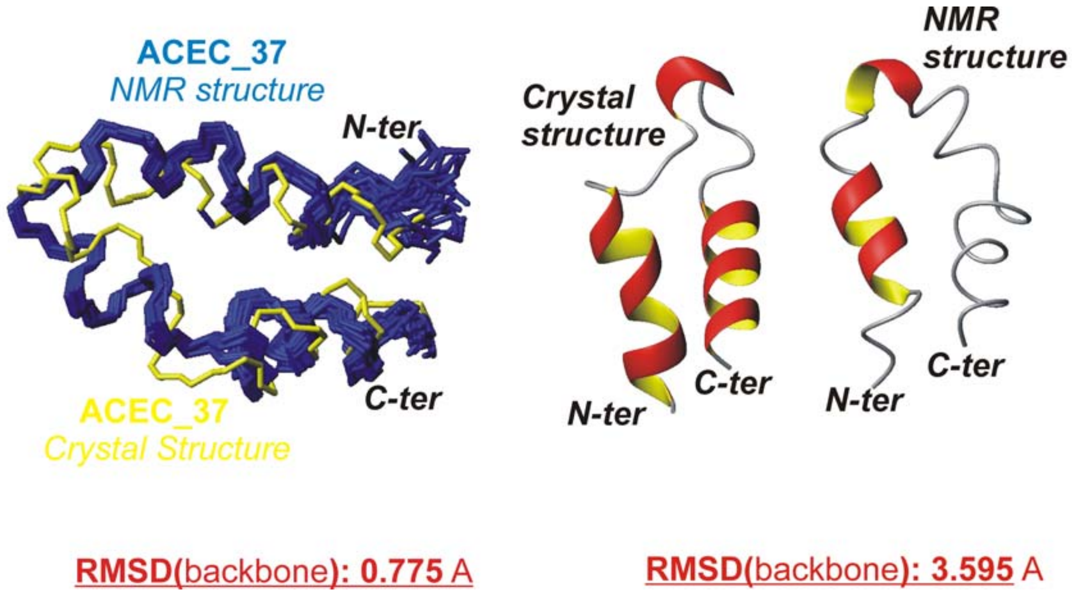

Figure S2. **Left:** Superimposition of Zn^{2+}-ACE_C(37) DYANA 20 best models calculated with NMR data in blue and the crystal structure of ACE_C in complex with lisinopril in yellow (pdb code:***1O86***). The calculated RMSD value is 0.775 Å for the backbone atoms. **Right:** Ribbon representation of the crystal structure of ACE_C in complex with lisinopril and the mean NMR calculated structure of Zn^{2+}-ACE_C(37). The calculated RMSD value is 3.595 Å for the backbone atoms.

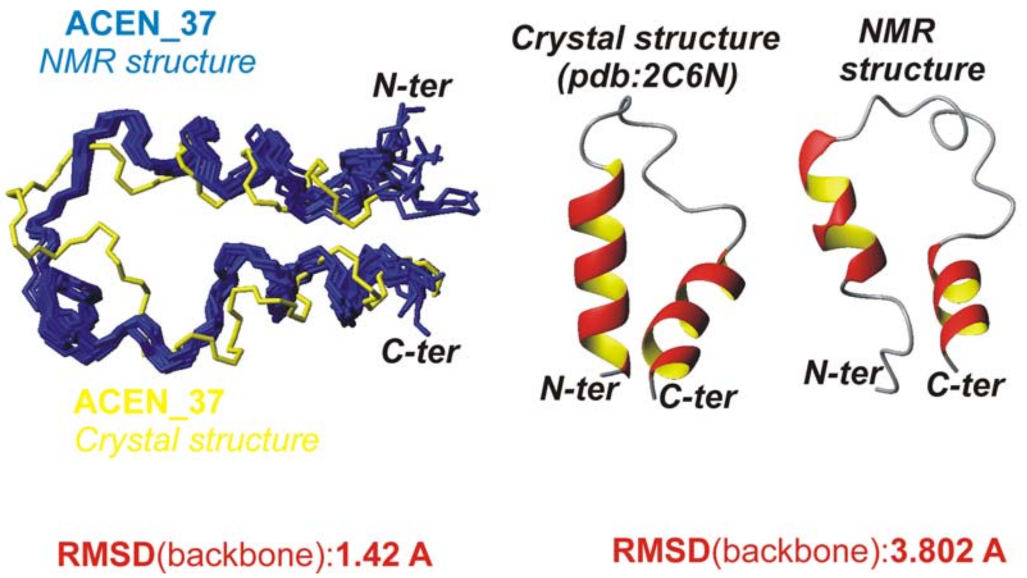

Figure S3: **Left:** Superimposition of Zn^{2+}-ACE_N(37) DYANA 20 best models calculated with NMR data in blue and the crystal structure of ACE_C in complex with lisinopril in yellow (pdb code:***2C6N***). The calculated RMSD value is 1.42 Å for the backbone atoms. **Right:** Ribbon representation of the crystal structure of ACE_N in complex with lisinopril and the mean NMR calculated structure of Zn^{2+}-ACE_N(37). The calculated RMSD value is 3.802 Å for the backbone atoms.
